# First Report of the Ticks *Haemaphysalis punctata* Canestrini et Fanzago, 1878, *Haemaphysalis parva* (Neumann, 1897) and *Dermacentor marginatus* (Sulzer, 1776) (Acari, Amblyommidae) from Humans in Lebanon

**DOI:** 10.2478/s11686-019-00160-7

**Published:** 2020-01-20

**Authors:** Martin Raad, Dany Azar, M. Alejandra Perotti

**Affiliations:** 1 Ecology and Evolutionary Biology Section, School of Biological Sciences, University of Reading, Whiteknights, Reading, RG6 6AS Berkshire UK; 2 Earth and Life Sciences Department, Faculty of Sciences II, Lebanese University, Fanar, Mount Lebanon Lebanon

**Keywords:** Tick, Acari, Amblyommidae, Lebanon, Pathogens, Mites

## Abstract

**Purpose:**

Knowledge on ticks infesting humans is scarce for the middle East. In this work, tick specimens (Acari: Amblyommidae) infesting humans in Lebanon were identified.

**Methods:**

Ticks that were found on humans were received in the Lebanese University, Faculty of Sciences. The specimens were preserved in alcohol for their further morphological identification.

**Results:**

Three tick species were identified: a red sheep tick *Haemaphysalis punctata* Canestrini et Fanzago, 1878, a Mediterranean ear tick *H. parva* (Neumann, 1897), and an ornate sheep tick *Dermacentor marginatus* (Sulzer, 1776); all isolated from human hosts.

**Conclusion:**

This is the first report of *Haemaphysalis punctata, H. parva* and *Dermacentor marginatus* infesting humans from Central and North Lebanon.

Ticks are ectoparasites infesting ruminants, livestock and humans all over the world, with records in many European and less frequently in Mediterranean countries i.e. Spain, Turkey, Romania, Italy, Iran, Palestine [1–4, 6, 8, 14, 16]. Reports are rare in the East Mediterranean region. In Lebanon, to the best of our knowledge, ticks infesting humans are still not reported, herein we present the first report of ticks collected on humans.

In late August 2017, a female ornate sheep tick was found on the head of a little boy at Qanat Bakish (Metn District; Mount Lebanon Governate, Central Lebanon), at 1800 m above sea level, 43 km North-East of Beirut. Based on the keys of Estrada-Peña et al. [10] and Mariana et al. [17], the female specimen (Fig. 1) was assigned to *Dermacentor marginatus* (Sulzer, 1776). Morphological, diagnostic features were confirmed: a small gnathosoma with small mouthparts; basis capituli with straight lateral margins; porose area shape narrow oval; palp article 2 posterior spur absent from dorsal surface; eyes always present and large; scutum of oval shape with white ornamentation; coxae I external and internal spurs gap located medium with the external spur slightly shorter than the interior spur.

**Fig. 1:**
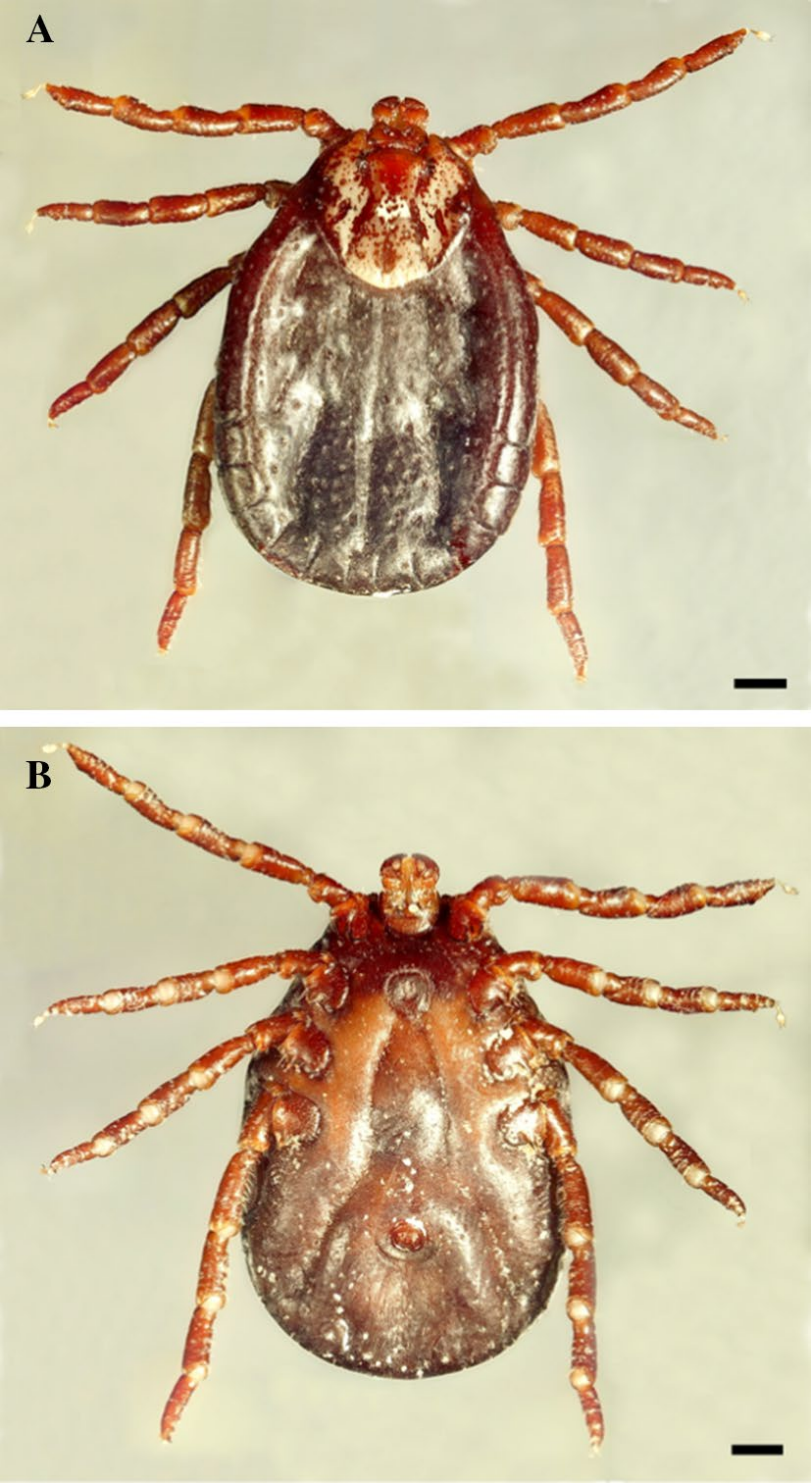
Adult female *Dermacentor marginatus*. **a** Dorsal view, scale bar 0.6 mm. **b** Ventral view, scale bar 0.6 mm

Later on, in January 2018, a red sheep tick was collected from the shoulder of an adult man in Mansourieh (Metn District; Mount Lebanon Governate, Central Lebanon). The infestation occurred at 250 m above sea level, 9 km South-East of Beirut. Based on the keys of Estrada-Peña et al. [10] and Hosseini-Chegeni et al. [14], the female tick was identified as *Haemaphysalis punctata* Canestrini et Fanzago, 1878 (Fig. 2a). There were no differences with diagnostic characters such as a small gnathosoma (Fig. 2b, red arrow), palp articles 2 broad but not too much extended (Fig. 3 blue arrow), basis capituli with straight lateral margins (Fig. 3 red arrow), scutum of oval shape (Fig. 3 green arrow), spiracular plates large and posterior to leg IV (Fig. 2b green arrow), and spurs of coxae IV distinct (Fig. 2b blue arrow).

**Fig. 2:**
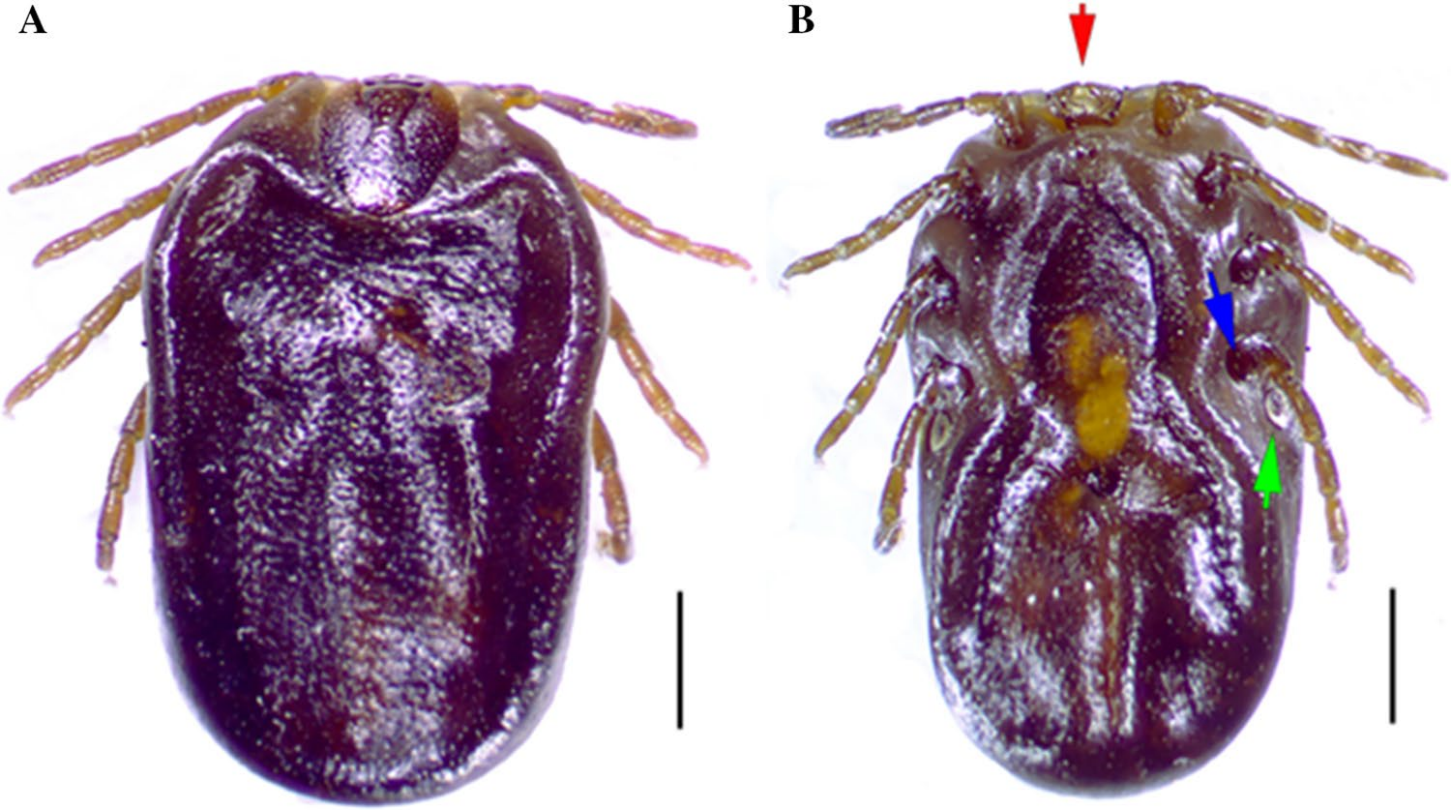
Adult female *Haemaphysalis punctata*. **a** Dorsal view, scale bar 1 mm. **b** Ventral view, scale bar 1 mm; red arrow indicates the small gnathosoma; green arrow indicates the large spiracular plates; blue arrow indicates the spurs of coxa IV

**Fig. 3:**
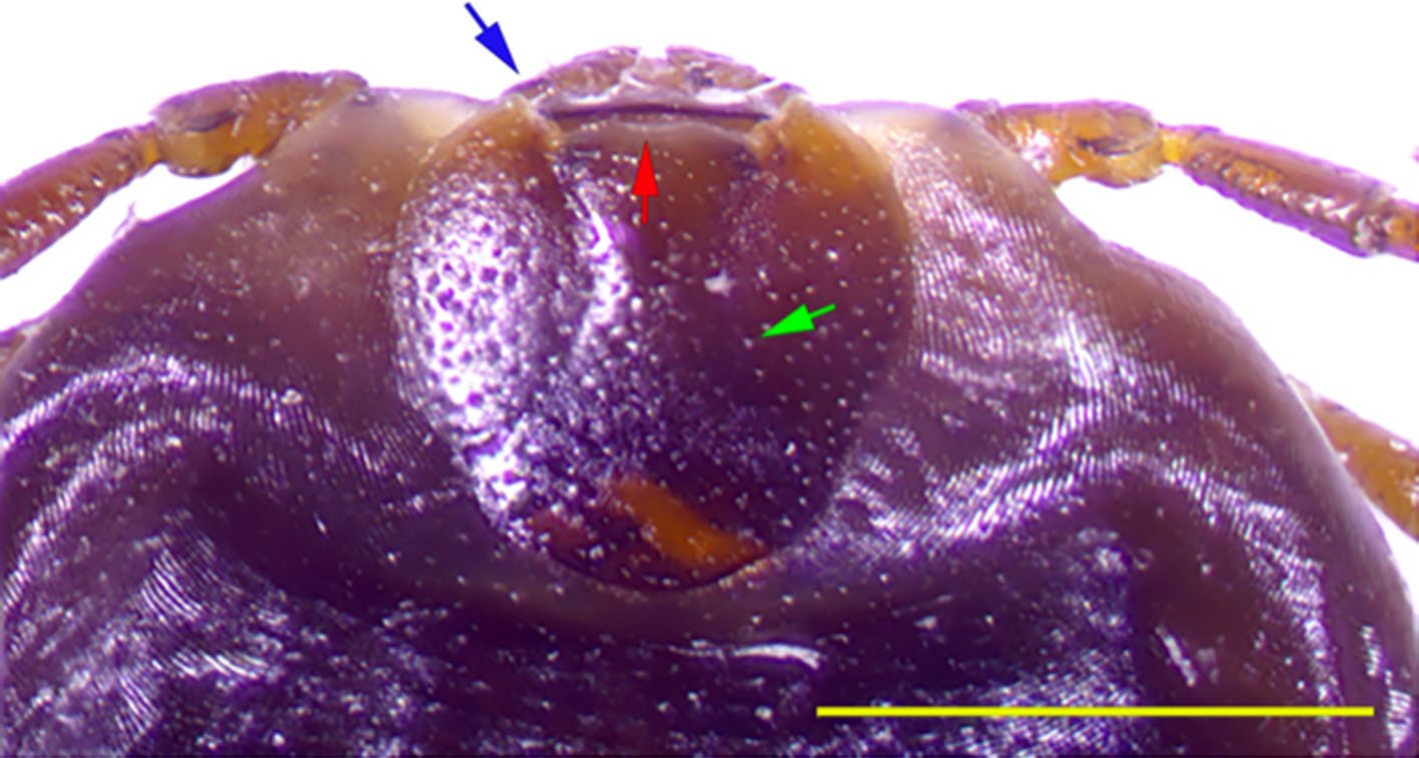
Detailed dorsal view of the apical part of adult female *Haemaphysalis punctata.* Red arrow indicates the *basis capituli*; green bracket indicates the oval *scutum*; and blue arrow indicates the palp segments. Scale bar 1 mm

Another species of *Haemaphysalis* sp. was found on October 4th 2018, collected on the hand of an adult male in Ehden Natural Reserve, North Lebanon, at 1250 m above sea level, 110 km North-East of Beirut. Using the keys of Hosseini-Chegeni et al. [14], a male was identified as a Mediterranean ear tick, *Haemaphysalis parva* (Neumann, 1897) (Fig. 4). The following features confirm the species: small gnathosoma; no pseudoscutum; palp article 2 without spur from dorsal surface and its lateral palpal segment is angled but not much extended; lateral palpal segment 2 width is wider than basis capituli; palpal segment 3 are straight not pincer-like; large spiracular plates are posterior to leg IV; anal grove has round shape instead of V shape; coxae I without gap in its spur; and coxae IV spur is short and not longer than coxae I–III.

**Fig. 4:**
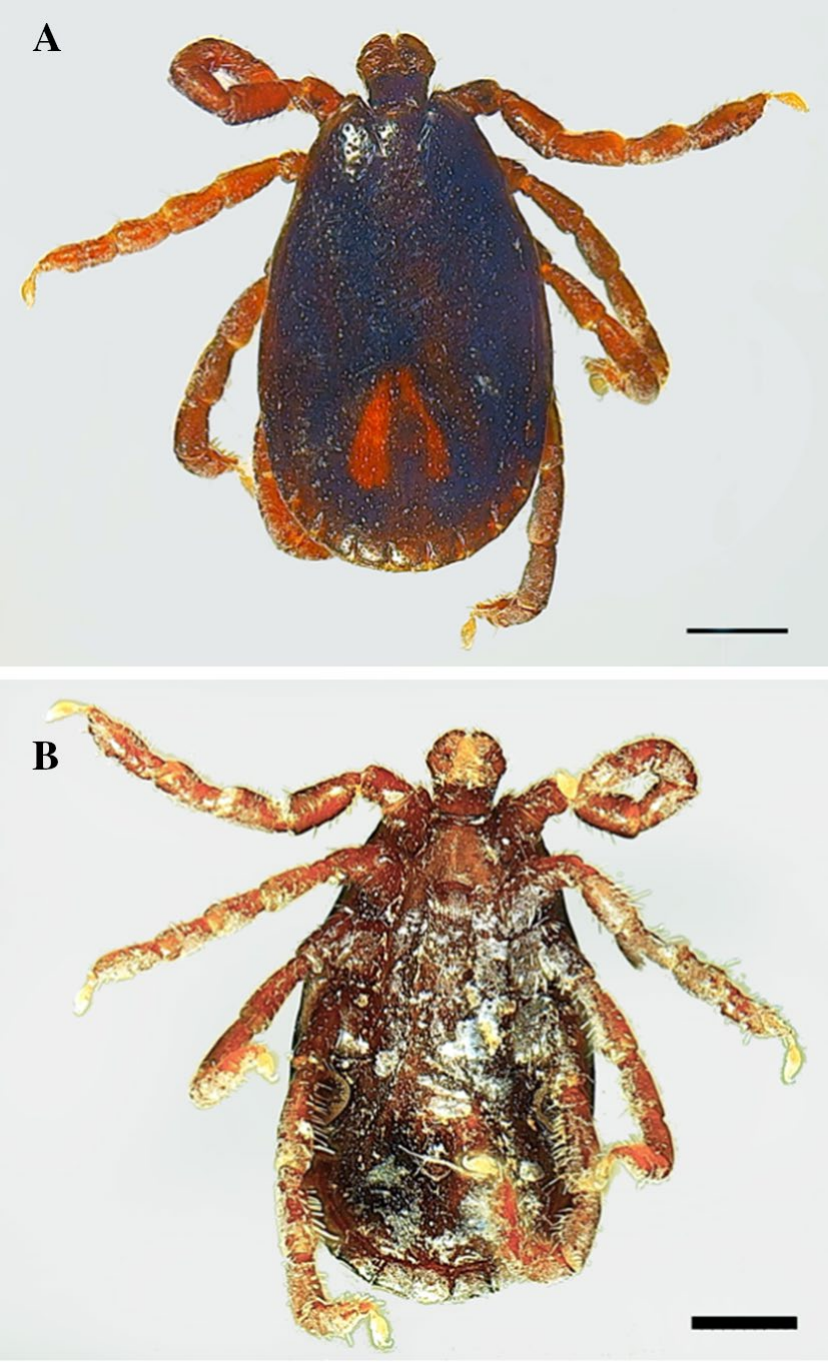
Adult male *Haemaphysalis parva*. **a** Dorsal view, scale bar 0.5 mm. **b** Ventral view, scale bar 0.5 mm

The red sheep tick *H. punctata* Canestrini et Fanzago, 1878 is a three-host tick with a life cycle lasting between one to three years (Estrada-Peña 2004). Ruminants and humans are its main hosts (Estrada-Peña 2004; [6, 8, 9]. In fact, *H. punctata* is known from many European, North African, Asian and Mediterranean countries—Palearctic realm (Estrada-Peña 2004; [1, 4, 5, 9, 11, 13, 16, 21]. Recently, many studies in the Middle East have reported *H. punctata* infesting humans; for instance, Turkey [6, 16], Palestine, Iraq [8], Iran [14], Sicily [3] and Israel [15]. Although *H. punctata* occurrence in Lebanon was lately reported by Dabaja et al. [7] and on ruminants, its finding on humans (mainly remains) has not been recorded yet from this country.

The ornate sheep tick *D. marginatus* (Sulzer, 1776) is a three-host tick with a complete life cycle of about one year. Its adult stage infests ruminants and dogs (Estrada-Peña 2004). Nevertheless, humans, rodents and birds are known hosts of its immature stages ([8, 9, 23, 26]. The species distribution includes the European, North African, and Mediterranean countries—the Palearctic region—as it is concentrated in environments of oak and pine vegetation, of optimal (thermophilic) requirements, especially when compared to other accompanying tick species i.e. *I. ricinus* and *H. punctata* (Estrada-Peña 2004; [1, 9, 13, 14, 16, 21, 23]. Even though several authors reported this tick infesting humans in Europe, Turkey, central Asia and Morocco (Estrada-Peña 2004, [3, 4, 8, 9, 22, 26], *D. marginatus* in Lebanon have occurred on ruminants from Baalbeck-El Hermel and Mount Lebanon Governates, but not on humans [7], until now.

Similarly, the Mediterranean ear tick *H. parva* (Neumann, 1897), a three-host tick, is found in the Mediterranean mountains and forests on high altitudes with high humidity level found in Turkey, Israel, Italy, Greece and other countries, hosting livestock and with human presence as well [6, 16, 18, 20, 21, 24, 25]. Parasitism of *H. parva* occurs during autumn, winter and spring with a peak in October and November [25]. While Bursali et al. [6] and Keskin et al. [16] have mentioned *H. parva* among the feeding ticks on humans in Turkey and Dabaja et al. [7] have cited many ticks found in Lebanon from livestock where 11.4% were *H. punctata*. Hence, no *H. parva* was cited from Lebanon till now. This is the first report of ticks found on humans in Lebanon, and of especial note is the finding of this rare species, *H. parva* (Neumann, 1897).

Ticks are generally causative agents of cross-infections in cattle, sheep, ruminants and humans. In the case of *H. punctata*, there is debate about its vector capability. Estrada-Peña et al. [10] and Dabaja et al. [7] suggested that *H. punctata* may transmit *Babesia* and *Theileria* species causing disease to humans [12]. Also, Andersson et al. [1] pointed out for tick-borne pathogens such as *Hepatozoon canis* related to *H. punctata* infesting animals in central Romania. Moreover, Estrada-Peña and Jongejan [9] and de la Fuente et al. [8] mentioned that Tick-Borne Encephalitis (TBE) virus and Crimean-Congo Hemorrhagic Fever (CCHF) virus may be transmitted by *H. punctata* as well.

Estrada-Peña et al. [10] and Blanda et al. [3] mentioned that *D. marginatus* can transmit *Rickettsia conori* and *R. aeschlimannii* to humans, respectively, causing tick typhus or boutonneuse fever. Parola and Raoult [22] claimed the presence of *Francisella tularensis* in *D. marginatus* ticks while infesting humans in Europe, North Africa and Central Asia. Further, Andersson et al. [1] and Keskin et al. [16] declared the occurrence of *Rickettsia raoultii* in one case, and *R. slovaca* and CCHF virus in another, via *D. marginatus* infesting humans in Romania and Turkey, respectively. Other publications stated that CCHF virus and *R. slovaca* were identified from *D. marginatus* ticks [9], as tick-borne infection, transmitted to humans [2].

Finally, Papa et al. [20] mentioned the potential of pathogen transmission by *H. parva* in Greece i.e. novel tick-borne phlebo viruses. Ozubek and Aktas [19] stated the occurrence of Ovine piroplasmosis and *Theileria annulata* in cattle from Turkey, found on ticks including *H. parva*. Keskin et al. [16] pointed out to the transmission of several tick-borne pathogens i.e. *Borrelia burgdorferi*, *Rickettsia hoogstraalii* and CCHF virus by *H. parva* tick while infesting humans.

Further studies concern the identification of pathogens carried by these three ticks species in Lebanon.
